# To Correspondents

**Published:** 1857-04

**Authors:** 


*To Correspondents.*

Not long since we received a letter from a subscriber at Plymouth Ind. enclosing two dollars. The writer, however, forgot to sign his name to it, and as there were two other subscribers in the same place we know not which should be credited with the money.

On the 28th of March, we received a letter through the post-office, which was accidentally lost before we learnt anything of its contents, or even the post-mark on it.

